# The Tölz Temporal Topography Study: Mapping the visual field across the life span. Part II: Cognitive factors shaping visual field maps

**DOI:** 10.3758/s13414-012-0279-y

**Published:** 2012-04-24

**Authors:** Dorothe A. Poggel, Bernhard Treutwein, Claudia Calmanti, Hans Strasburger

**Affiliations:** 1 Generation Research Program (GRP), Ludwig Maximilian University Munich, Human Science Center, Bad Tölz, Germany; 2 Dept. of Medical Psychology and Medical Sociology, Georg August University Göttingen, Göttingen, Germany; 3 IuK, Ludwig-Maximilian University Munich, Munich, Germany; 4 Hanse-Wissenschaftskolleg Institute of Advanced Study, Lehmkuhlenbusch 4, 27753 Delmenhorst, Germany

**Keywords:** Temporal processing, Visual field maps, Alertness, Divided attention, Visual search, Aging

## Abstract

Part I described the topography of visual performance over the life span. Performance decline was explained only partly by deterioration of the optical apparatus. Part II therefore examines the influence of higher visual and cognitive functions. Visual field maps for 95 healthy observers of static perimetry, double-pulse resolution (DPR), reaction times, and contrast thresholds, were correlated with measures of visual attention (alertness, divided attention, spatial cueing), visual search, and the size of the attention focus. Correlations with the attentional variables were substantial, particularly for variables of temporal processing. DPR thresholds depended on the size of the attention focus. The extraction of cognitive variables from the correlations between topographical variables and participant age substantially reduced those correlations. There is a systematic top-down influence on the aging of visual functions, particularly of temporal variables, that largely explains performance decline and the change of the topography over the life span.

## Introduction

### Background and motivation of the study

This second part of the Tölz Temporal Topography Study describes how cognitive variables, particularly measures of visual attention, are related to the psychophysical data presented in Part I. The study is based on an extensive data set of visual field maps and cognitive variables acquired within the Vision Lab of the Generation Research Program at Bad Tölz, with the goal of describing and analyzing the topography of visual performance and its changes over the life span. For reasons of length, the study is presented in two parts. Part I describes and interprets topographical variables of temporal processing and light detection, their relationship to each other, and their variation over the life span. In Part II, cognitive variables, particularly visual attention measures, are related to the psychophysical data presented in Part I.

A main result of Part I was the dissimilarity of topographies and their different change with age. Time-related parameters fell into two groups, one reflecting processing speed (reaction time [RT]), and the other reflecting sensitivity or signal-to-noise ratio (double-pulse resolution [DPR]). The increase of RT over age happened rather uniformly over all visual field positions. DPR was unrelated to RT and, instead, resembled perimetric luminance thresholds in both topography and aging. Particularly for DPR thresholds, the periphery of the visual field showed a stronger reduction of performance than did foveal field positions, but interindividual variation was high, and age was not a good predictor of performance. Hence, other factors that co-vary with age—such as cognitive performance parameters—may influence visual maps and their change over the life span.

### Cognitive influences on visual performance

Visual information traveling from the retina to the primary visual cortex and beyond is processed and shaped by the structure and functional specialization of the visual system. In addition to bottom-up processes, however, visual processing at lower stages in the pathway is profoundly influenced by top-down modulation originating from higher visual areas and from networks representing cognitive functions such as such as attention and memory (Bressler, Tang, Sylvester, Shulman, & Corbetta, 2008; Noesselt et al., 2002; Soto, Hodsoll, Rotshtein, & Humphreys, 2008). Attention, in particular, has been shown to modify behavioral performance and neuronal activation as far down as V1 and even subcortical brain regions (Giesbrecht, Woldorff, Song, & Mangun, 2003; Kastner, Pinsk, De Weerd, Desimone, & Ungerleider, 1999; Mangun, Buonocore, Girelli, & Jha, 1998; Mangun & Fannon, 2007; Natale, Marzi, Girelli, Pavone, & Pollmann, 2006; Schneider & Kastner, 2009). Cognitive influences on visual processing vary with stimulus characteristics, but also with task demands (Eimer, Kiss, Press, & Sauter, 2009; Toth & Assad, 2002; Travers & West, 2008).

As was shown in Part I of this study, visual performance is distributed across the visual field in a characteristic way for a specific visual function. The center and periphery of the visual field show large differences of visual performance, usually a lower performance in the periphery than in the center of the visual field (see the Introduction and Discussion in Part I). Focusing attention at a specific location within the visual field can change these performance maps in healthy observers (Carrasco, 2006), as well as in patients with partial vision loss (Poggel, Kasten, Müller-Oehring, Bunzenthal, & Sabel, 2006). The effectiveness of focusing attention depends, among many other factors, on the size of the attention focus; the larger the size of the attended area, the lower is the processing capacity and, hence, the lower is the gain from focusing attention at that location (Castiello & Umiltà, 1990).

### Aging of visual and cognitive performance

A large number of studies have shown a decline of visual performance in the elderly (Brabyn, Schneck, Haegerstrom-Portnoy, & Lott, 2001; Fiorentini, Porciatti, Morrone, & Burr, 1996; Haegerstrom-Portnoy, Schneck, & Brabyn, 1999; Owsley, 2011; Rubin et al., 2001; Schneck, Haegerstrom-Portnoy, Lott, Brabyn, & Gildengorin, 2004; West et al., 2002). This particularly applies to any type of speeded processing, like the measurement of RTs or other indicators of temporal-information processing (Falkenstein, Yordanova, & Kolev, 2006; Haier, Jung, Yeo, Head, & Alkire, 2005). Age-related deterioration of speeded performance has been related to functional and structural changes in the brain (Birren & Fisher, 1995; Eckert, Keren, Roberts, Calhoun, & Harris, 2010; Falkenstein et al., 2006; Haier et al., 2005; Spear, 1993).

Cognitive processes change over the life span. A typical pattern is an increase of cognitive performance until early adulthood, caused by maturation of the nervous system, followed by a decline toward senescence (Craik & Bialystok, 2006). While this prototypical development is not found for all functions (e.g., not for crystallized intelligence), particularly any measures of speeded or fluid performance tend to follow that pattern (e.g., fluid intelligence or speeded attention (Aizpurua & Koutstaal, 2010). Attentional capacity is also reduced with increasing age (Groth & Allen, 2000; Madden, 2007). This has an indirect influence on the size of the attention focus that can be maintained for efficient visual processing (Groth & Allen, 2000; Pesce, Guidetti, Baldari, Tessitore, & Capranica, 2005).

While there is an extensive literature on the effects of visual performance decline over age on cognitive functions (Groth & Allen, 2000; Pesce et al., 2005), there is, to our knowledge, no systematic study showing the influence of cognitive aging on visual function and visual field maps (cf. Owsley, 2011; Werner, Peterzell, & Scheetz, 1990).

### Hypotheses

Given these interconnections of visual processing, cognitive factors, and aging, it is likely that the changes of visual field maps over the life span observed in Part I of this study are caused not only by the deterioration of the optical apparatus and neural structures in the visual pathway, but also by higher visual and cognitive functions. As was mentioned above, the size of the attention focus is relevant for visual processing. In addition, the individual’s general alertness and ability to divide attention between different locations or sources of information are prerequisites for efficient handling of visual tasks. The ability to shift attention covertly (as assessed, for instance, in the Posner (1980) paradigm) and overtly (visual search) is also likely related to visual performance—in particular, for temporal variables that are the main focus in this study.

We therefore hypothesized the following:

1. Cognitive functions—in particular, attentional functions—influence the topography of visual functions, especially with respect to variables of temporal processing.
2. Cognitive factors, particularly attentional functions, can differentially influence the shape of visual field maps, depending on the cognitive task demands involved in measuring a particular visual variable.
3. Due to their aging, cognitive functions exert a differential influence on topographical tests of visual performance at different stages in life.
4. The shape of a visual field map and its variation across the life span is the result of an interaction of optical and lower-level visual functions and cognitive processes.

## Method

### Sample

The sample consisted of 95 paid volunteers (26 of them male) between 10 and 90 years of age (mean age: 47.8 years; see Table 1, Part I). All observers had normal or corrected-to-normal vision. Severe dementia, impairments of attention or other cognitive functions, and depression or other psychiatric disorders, as well as brain lesions and the presence of visual impairment at any level of the visual pathway, were exclusion criteria. In particular, the older participants were asked about potential ophthalmic diseases, and we inquired about the most recent eye exam to ascertain that participants were clinically inconspicuous. All observers (or their parents for minors) gave their informed consent for participation. The study design had been approved by the ethics committee of the Ludwig-Maximilian University, Munich, Germany, and testing procedures were in accordance with the tenets of the Declaration of Helsinki.

### Test conditions and general setting

Testing took place under standardized conditions for all observers. Total testing time was approximately 7 h per participant, with some interindividual variation due to the different duration of the thresholding tests. With very few exceptions, the tests were performed in several sessions of 1.5- to 3.5-h duration, usually over a period of 1–2 weeks. All the variables mentioned in Parts I and II were examined in all participants; that is, for participants, the separation into a basic vision and a cognitive part of the experiment was not apparent.

Participants were allowed to take breaks at any time to avoid excessive fatigue. The experimenter was present throughout the test session(s), observed the participant’s performance and gaze position in a mirror, and interrupted the test when he or she appeared tired.

### Topographical variables

Thresholds of double-pulse resolution (DPR) were measured using an apparatus and psychophysical technique developed by Treutwein (1989, 1995, 1997); Treutwein & Rentschler (1992). In an adaptive, nine-alternative forced choice task, the observer identified the noncontinuous stimulus in an array of nine stimuli so that the minimum detectable temporal gap between two light pulses was determined. Testing was performed in a darkened room with 30-cm viewing distance (see Fig. 1 of Part I). Each observer performed ten blocks of trials with a specific eccentricity of the peripheral stimuli (i.e., ring radius) set for each block. Each eccentricity (2.5°, 5°, 10°, 15°, and 20°) was tested twice. Test speed and duration were controlled by the observer responding in a self-paced manner.

RT maps were assessed with a high-resolution (474 positions) computer-based campimetric test (Nova Vision, Magdeburg, Germany) under the same testing conditions (darkened room, 30-cm viewing distance) as those for DPR threshold measurement (display size: ± 27° × ± 22.5°; cf. Fig. 2 of Part I). Participants reacted to the presentation of small, suprathreshold white-light stimuli on a dark background by pressing a key on the computer keyboard. Total duration of this test was 20 min. RTs corresponding to the visual field positions for DPR thresholds were used for analysis.

For perimetric testing, observers were examined with the G2 program implemented on the Octopus 101 Perimeter (Interzeag/ Haag-Streit, Wedel, Germany) to determine luminance detection thresholds within the central 30° of the visual field. Tests were performed separately for each eye, with a test duration of 10–12 min per eye. Fixation was controlled by an infrared-sensitive camera integrated into the sphere. The observer pressed a response button upon detecting a stimulus presented in the periphery (background luminance: 10 cd/m²). The G2 test uses a simple adaptive procedure for the independent variation of stimulus luminance at the 59 test positions and includes catch trials.

Contrast thresholds for the recognition of characters were tested with the software R_Contrast (Strasburger, 1997), which uses ML-PEST as the thresholding algorithm (Harvey, 1997). The general setting was the same as that for the computer-based RT measurement. Contrast thresholds were determined for the central (foveal) stimulus position and on the horizontal and vertical meridians at ± 10° eccentricity. On a gray background (36 cd/m^2^), the digits from 0 to 9 were presented at 2.4° visual angle height (viewing distance: 43 cm) for 100 ms each in randomized order. Initially, the digits were white, and the contrast to the background was reduced with every correct answer and, for an incorrect response, was increased again. The answers were given verbally, and the experimenter entered the responses into the software using the computer keyboard. A run was ended when the (estimated) 95% confidence interval of the Michelson contrast threshold value undercut 0.2 log units. Duration of a test run was 5–10 min.

### Saccadic exploration

We examined the search field—that is, the distribution of speed and accuracy of saccadic eye movements toward peripheral targets—with a subroutine of the Nova Vision test battery. The basic setting was the same as that for the RT mapping described above.

Participants were instructed to keep their eyes on the fixation mark in the center of the screen and then to make eye movements toward a stimulus in the periphery of the visual field that was presented simultaneously with an acoustic signal. The stimulus was the digit “2,” “3,” or “8,” presented in random order at 284 positions in the visual field. Observers responded by pressing one of three keys on the computer keyboard (arrow left, arrow down, arrow right) without taking their gaze off the screen. After the response, they returned their gaze to the fixation point. Saccadic RTs for each position were registered by the program and plotted in the same way as the RTs from the computer-based visual field test.

### Attentional variables

Three functions of attention were assessed by a computer-based test battery TAP (“Test Battery for Attentional Performance,” a standard tool for assessment of attentional functions in German-speaking countries [Zimmermann & Fimm, 1995]). In the *alertness* subtest, a central visual stimulus was presented with versus without an acoustic cue preceding its presentation by 100 ms. In cued as well as in uncued runs, the observer’s task was to press a key as quickly as possible in response to the appearance of the stimulus. Cued and uncued RTs and their difference were registered. Four runs were performed in an A–B–B–A design. The test took approximately 5 min for all four runs.

*Divided attention* was examined with a dual visual–auditory subtest. The visual task consisted of monitoring a display of 8 × 8 positions that were filled by either dots or crosses. The stimulus pattern changed every 1.5 s and, at random intervals, formed a square configuration of crosses on the screen. This was the critical visual stimulus to be detected by the observer and indicated by a keypress. Simultaneously with the visual task, a series of alternating high and low tones was presented, and the observer was instructed to monitor the tones for a break in the sequence—that is, two high tones or two low tones—which was the critical acoustic stimulus. RTs to the critical visual and acoustic stimuli, as well as hits and misses, were recorded. Total test duration was approximately 10 min.

To test the ability to covertly shift attention to a visual field location indicated by a cue and the costs and benefits of attending to a cued visual field location (Posner, 1980), a third subtest of the TAP was employed (Posner paradigm). The observer fixated a central fixation mark and made a speeded response to a cross appearing at a fixed position in either the left or the right visual field. Before the presentation of a target, an arrow above the central fixation mark indicated the target position with 80% validity. RTs were measured for valid trials (target presented at the position indicated by the arrow) and invalid trials (target presented at the position opposite to the one indicated by the cue). The RT difference between the two conditions was registered as cue benefit. Total test duration was approximately 10 min.

### Data analysis

In a first step, raw data were analyzed separately for each test. Parametric tests (*t*-test and GLM/ANOVA) were used for the comparison of means. Correlations between test parameters were computed using Pearson’s *r*. Additionally, the influence of cognitive variables was extracted from the correlation of age with the topographical/temporal variables by using the partial correlation technique to explore the influence of cognitive factors on visual field maps. We calculated the common variance (*R* ^2^) of two variables from the original correlation value, as well as the common variance (*R*_part_^2^) remaining after the cognitive factor had been extracted as the square of the partial correlation. The absolute amount of extracted variance for a specific cognitive variable was then determined as the difference between *R* ^2^ and *R*_part_^2^. The relative amount of extracted variance was further calculated as (*R* ^2^ − *R*_part_^2^)/*R* ^2^ (e.g., 100% relative extracted variance means that all the common variance was extracted).

Data were extracted from the RT maps only at the DPR test positions. In the perimetric maps, luminance threshold values at the DPR-grid positions were determined by interpolation, because the stimulus positions in perimetry did not correspond to those of the DPR test.

All statistical testing was performed using SPSS software (Version 12.0, Chicago, IL). The alpha level was set to .05 (two tailed). For a detailed description of the analysis of the topographical variables, see Part I.

## Results

### Topographical variables

Results on the topographical variables from Part I (DPR, RT, perimetry, R_Contrast) are briefly summarized here for their comparison with the cognitive variables. The figures in Part I illustrate the effects described below.

Thresholds of DPR increased toward the periphery of the visual field, with a steep increase from the center to 2.5° eccentricity and a shallow, steady rise of thresholds beyond 5°. Age had a significant effect on mean DPR thresholds: The best performance was observed in participants between 20 and 30 years of age; up to the age of 60, there was hardly any increase of mean thresholds, but we observed a sharp increase in the 70s and 80s. The form of the DPR threshold maps changed over the life span; that is, there was an interaction between age and eccentricity, with older participants showing a steeper incline of thresholds beyond 5° of eccentricity (see Figs. 5 and 6, Part I); that is, there was a stronger reduction of performance in the periphery than in the center of the visual field for older observers.

Simple visual RTs showed a shallow, evenly distributed increase from the center to the periphery of the visual field. The aging effect on mean RTs was significant, with best performance in the 30s and a noticeable increase after 60 years. The (overall) interaction of age and eccentricity missed significance; the increase of RTs toward the periphery was steeper in the two oldest age groups, however.

Our results on perimetric thresholds of light detection replicated earlier findings of increasing thresholds toward the periphery. Mean luminance thresholds were significantly affected by age: The youngest participants showed the best performance, and the three oldest age groups had increased thresholds, as compared with the remainder of the sample. For perimetric performance the effect of age was more systematic and much stronger than for the other variables examined in this study. In contrast to DPR thresholds, however, there was no significant interaction between the aging and eccentricity effect. Only singular elderly participants showed a disproportionately stronger increase of perimetric thresholds in the periphery than did the other age groups.

Mean contrast thresholds for character recognition (R_Contrast) were higher at 10° eccentricity than in the fovea. Mean thresholds over all tested positions increased slightly but significantly with age, and there was a significant interaction of age and eccentricity.

Thus, the topographical variables of temporal and light/contrast processing showed different forms of visual field maps and also differing aging processes.

### Correlation of topographical and cognitive variables

Table 1 shows a summary of all correlations between cognitive and topographical variables. Almost all correlations were medium to high, and particularly, the ability to shift attention—overtly (saccadic RTs) or covertly (Posner paradigm)—had a close connection with the topographical variables.

**Table 1:** Pearson correlations of topographical variables with cognitive parameters

Subtest	Variable	DPR Thresholds	Simple RT in Campimetric Test	Perimetric Thresholds	Character Recognition Contrast Thresholds (R_Contrast)
TAP Alertness	overall mean RT	.35 (.001)	.35 ( < .001)	−.25 (.015)	.32 (.002)
	mean RT difference cued−uncued	−.19 (.061)	−.07 (.534)	.13 (.212)	−.09 (.387)
TAP Divided Attention	mean overall RT	.34 (.001)	.30 (.004)	−.20 (.055)	.24 (.019)
	mean number of correct detections	−.39 (<.001)	−.44 ( < .001)	.07 (.488)	−.18 (.089)
	mean visual RT	.34 (.001)	.14 (.182)	−.26 (.013)	.22 (.033)
	mean auditory RT	.34 (.001)	.27 (.009)	−.07 (.513)	.21 (.048)
TAP Posner Paradigm	mean RT valid trials	.54 (<.001)	.46 ( < .001)	−.39 ( < .001)	.43 ( < .001)
	mean RT invalid trials	.50 (<.001)	.51 ( < .001)	−.30 (.004)	.42 ( < .001)
	cue benefit (mean difference valid−invalid)	.43 (<.001)	.49 ( < .001)	−.21 (.046)	.37 ( < .001)
Saccadic exploration	mean search RT	.62 (<.001)	.32 (.002)	−.47 ( < .001)	.49 ( < .001)

TAP = test of attentional performance; *p* values in parentheses

### Topographical attention effect in DPR threshold maps

DPR thresholds were always measured simultaneously at all nine positions in each block. This included the central (foveal) position, which remained constant over all blocks of testing. Since the physical position of the central test location did not change, one would expect constant foveal DPR thresholds, independent of the variation of the peripheral test locations. However, as we have shown earlier (Fig. 4 of Poggel, Treutwein, Calmanti, & Strasburger, 2006), the central DPR thresholds increase with the radius of the display. The pattern and the average rate of increase were almost exactly the same as those for the increase of DPR thresholds at the peripheral positions. Since the stimulus characteristics and all other test conditions were identical, we interpreted the increase of central thresholds with increasing display size as the effect of attentional processing capacity being distributed over a larger area (see Poggel, Treutwein, et al., 2006, for a detailed discussion).

Interestingly, while the attention effect at the central stimulus location was pronounced and highly significant for the total group of observers, it was absent for the youngest participants (10–20 years) and was very small in participants between 20 and 40 years of age (see Fig. 7 in Poggel, Treutwein, et al., 2006). For all other age groups—that is, from 40 to 90 years—the increase of central DPR thresholds with increasing radius of the test display was large and highly significant. The attention effect was particularly pronounced for the oldest age group of 80- to 90-year-old participants, who also showed the steepest increase of DPR thresholds toward the periphery.

### Saccadic exploration (overt attention)

The mean RT in the saccadic exploration test (mean over all observers and test positions ± *SEM*) was 851.4 ms ± 17.5 ms. There was a significant increase of search times over the life span, with the best performance in the 20- to 29-year-old participants (697.5 ± 28.7 ms) and the lowest in the oldest group [1,216.8 ± 66.9 ms; analysis of variance over age groups: *F*(7, 85) = 13.0, *p* < .001, *η* ^2^ = .52]. The effect of age was also reflected in the substantial correlation of mean search time and observer age, *r*(93) = .64, *p* < .001.

In the following, attentional variables were extracted from the correlations of the main topographical variables with age, to explore their influence on visual field maps (cf. the corresponding section on each variable below). When saccadic RTs were extracted, the age correlations were substantially reduced for all topographical variables except the campimetric RT map (Table 2), the effect being most pronounced for DPR.

**Table 2:** Pearson correlations of topographical variables with age and corresponding partial correlations where saccadic exploration time is partialled out

Variable	Correlation With Age:	Partial Correlation With	Extracted Variance
	Pearson’s *r* (*p*)	Age *r* (*p*), Saccadic Reaction	Absolute: R^2^ − R_part_^2^
	*R* ^2^	Times Partialled Out	Relative: (R^2^ − R_part_^2^) /R_2_
DPR thresholds	.62 ( < .001)	.35 ( = .001)	26.2%
	38.4%		68.2%
RT campimetric test	.16 (.118)	−.05 ( = .628)	2.3%
	2.6%		88.5%
Perimetric thresholds	−.68 ( < .001)	−.55 ( < .001)	16.0%
	46.2%		34.6%
R_Contrast	.50 ( < .001)	.31 ( = .002)	15.4%
	25.0%		61.6%

### Nontopographical attentional variables from the TAP

#### Alertness

The average RT in the alertness subtest of the TAP over all observers and trials was 267.7 ms (± 6.1). Without an acoustic cue, observers took an average of 272.1 ms (± 6.0) to respond to the visual stimulus, while on trials with an acoustic cue, the average RT amounted to 266.8 ms (± 7.0). Thus, there was an overall advantage of 5.3 ms (± 3.6) of cued over uncued trials.

Overall, RTs in the TAP Alertness Test increased over the life span [ANOVA, *F*(7, 87) = 2.21, *p* = .041, *η* ^2^ = .15], but not monotonously. The best performance was obtained in participants between 30 and 39 years of age, and the lowest in the oldest age group (80–90 years). The same pattern was found for uncued and for cued RTs developing over the life span. Although the correlations of RTs in the TAP Alertness Test with age were all significant, they were not substantial (Table 3) and were considerably lower than the correlations of search times with age.

**Table 3:** Correlations of attentional subtests in the TAP with age (*N* = 95)

TAP Subtest	TAP Variable	Correlation With Age: *r*(93)	*p*
Alertness	Median RT all trials	.32	< .001
	Median RT uncued trials	.33	< .001
	Median RT cued trials	.28	.006
Divided attention	Median RT all trials	.22	.036
	Number of correct resp.	−.19	.063
	Median visual RT	.18	.075
	Median auditory RT	.20	.054
Covert attention shift (Posner paradigm)	Median RT valid trials	.55	< .001
	Median RT invalid trials	.47	< .001
	Difference (cueing benefit)	.37	< .001

When RTs from the TAP Alertness Test were extracted from the correlations of the main topographical variables with age, the common variance of age with DPR thresholds, with campimetric RT, and with perimetric thresholds, respectively, was somewhat reduced (8%–10% extracted variance), while R_Contrast remained largely unchanged (Table 4).

**Table 4:** Correlations of topographical variables with age and partial correlations where response times in the TAP Alertness Test are extracted

Topographic Variable	Correlation With Age:	Partial Correlations—Extracted Variables	Alertness Median RT	Alertness Median RT Difference Cued−Uncued Trials
	*r* (*p*)		All Trials	
	*R* ^2^			
DPR thresholds	.65 ( < .001)	*r*_part_ (*p*)	.57 ( < .001)	.63 ( < .001)
	42.3%	% absolute variance extracted	9.8%	2.6%
		% relative variance extracted	23.2%	6.1%
RT campimetric test	.30 (.003)	*r*_part_ (*p*)	.05 (.610)	.16 (.120)
	9.0%	% absolute variance extracted	8.8%	6.4%
		% relative variance extracted	97.8%	71.1%
Perimetric thresholds	−.71 ( < .001)	*r*_part_ (*p*)	-0.65 ( < 0.001)	-0.68 ( < 0.001)
	50.4%	% absolute variance extracted	8.2%	4.2%
		% relative variance extracted	16.3%	8.3%
R_contrast thresholds	.47 ( < .001)	*r*_part_ (*p*)	.45 ( < .001)	.50 ( < .001)
	22.1%	% absolute variance extracted	1.8%	−2.9%
		% relative variance extracted	8.1%	−4.5%

#### Divided attention

The median RT of all observers in the divided attention task amounted to 698.9 ms (± 10.6), with 878.4 ms (± 14.9) for the visual subtask and 565.7 ms (± 12.1) for the auditory subtask. The average number of correct detections of critical events (both modalities) was 27.8 (± 0.5).

Over the life span, overall, RTs in the TAP Divided Attention Test significantly increased [ANOVA: *F*(7, 87) = 4.04, *p* = .001, *η* ^2^ = .25], reaching from 634.0 ms (± 24.2) for the group of 20- to 29-year-olds to 836.1 ms (± 41.9) for the group of 80- to 90-year-olds. Similarly, there was a reduction of the number of correctly identified critical events with age [ANOVA: *F*(7, 87) = 3.43, *p* = .003, *η* ^2^ = .22], ranging from 29.4 (± 1.4) for participants in their 30s to 20.2 (± 1.9) for participants in their 80s. Similarly, the visual RTs [ANOVA: *F*(7, 87) = 2.79, *p* = .011, *η* ^2^ = .18] and auditory RTs [ANOVA: *F*(7, 87) = 1.85, *p* = .088, *η* ^2^ = .13] increased over the life span, with optimal performance for participants in their 20s and lowest performance in the oldest age group.

Correlations of parameters of divided attention with age were relatively low, and only some became significant (Table 3).

We extracted the influence of divided attention from the correlation between age and the topographical variables and found, similar to the influence of alertness, some reduction of the common variance for the topographical variables (up to a quarter, relative to the original shared variance), again with the exception of R_Contrast (Table 5).

**Table 5:** Correlations of topographical variables with age and partial correlations where response times in the TAP Divided Attention Test are extracted

Topographic Variable	Correlation With Age:	Partial Correlations—Extracted Variables	Divided Attention Median RT, All Trials	Divided Attention, mean Number of Correct Detections
	*r* (*p*)			
	*R* ^2^			
DPR thresholds	.65 ( < .001)	*r*_part_ (*p*)	.59 ( < .001)	.60 ( < .001)
	42.3%	% absolute variance extracted	7.4%	6.3%
		% relative variance extracted	17.5%	14.9%
RT campimetric test	.30 (.003)	*r*_part_ (*p*)	.05 (.610)	.09 (.403)
	9.0%	% absolute variance extracted	8.8%	8.2%
		% relative variance extracted	97.8%	91.1%
Perimetric thresholds	−.71 ( < .001)	*r*_part_ (*p*)	−.66 ( < .001)	−.68 ( < .001)
	50.4%	% absolute variance extracted	6.9%	4.2%
		% relative variance extracted	13.7%	8.3%
R_Contrast thresholds	.47 ( < .001)	*r*_part_ (*p*)	.47 ( < .001)	.48 ( < .001)
	22.1%	% absolute variance extracted	0%	0%
		% relative variance extracted	0%	0%

#### Covert attention

The median RTs of all observers in the covert attention task (Posner paradigm) were 305.4 ms (± 6.8) for valid and 699.8 ms (± 16.4) for invalid trials. The mean difference between invalid and valid trials amounted to 394.1 ms (± 10.6). RTs on valid trials increased with age [ANOVA: *F*(7, 87) = 8.16, *p* < .001, *η* ^2^ = .40; best performance: 258.0 ms (± 13.9) in the 20s group; lowest performance: 403.6 ms (± 24.0) in the 80s group], as did RTs for invalidly cued trials [ANOVA: *F*(7, 87) = 6.14, *p* < .001, *η* ^2^ = .34; best performance: 584.2 ms (± 34.6); lowest performance: 896.4 ms (± 60.0)]. The greatest benefit of valid over invalid cues with respect to RT differences was found in the oldest group (80–90 years: 492.8 ± 40.9 ms). Participants in their 20s, although showing the fastest responses on both valid and invalid trials, profited least from the cue (20–30 years: 326.2 ± 23.6 ms). The absolute increase of the cueing benefit over the life span was significant [ANOVA: *F*(7, 87) = 4.14, *p* = .001, *η* ^2^ = .25]. However, that increase could be due to a multiplicative effect of overall slowed RT with increasing age: When the relative increase of the cueing benefit was calculated using the ratio (RT_valid_ − RT_invalid_)/RT_mean_, there was no significant change over the life span [ANOVA: *F*(7, 85) = 1.31, *p* = .256, *η* ^2^ = .10]. Of all the subtests of the attention test battery, the variables of the Posner paradigm showed the highest correlations with observer age (Table 2).

Again, the variables of covert attention were extracted from the correlation between the topographical variables and age. We found that the common variance of age with DPR thresholds was, in absolute values, the one most strongly affected by extracting covert attention variables, with more than half of the shared variance extracted in relative values (Table 6). Campimetric RT’s moderate correlation with age was almost completely mediated by covert attention variables; that is, close to 100% of the originally shared variance was extracted. The extraction of covert attention variables also had a large effect on perimetric thresholds and on R_Contrast (Table 6).

**Table 6:** Correlations of topographical variables with age and partial correlations where response times in the TAP covert attention test (Posner paradigm) are extracted

Variable	Correlation With Age:	Partial Correlations—Variables Extracted		
	*r* (*p*)	Posner Median RT, Valid Trials	Posner Median RT, Invalid Trials	Posner Cue Benefit (Mean RT Difference Valid−Invalid Trials)
DPR thresholds	.65 (< .001)	.45 ( < .001)	.49 ( < .001)	.54 ( < .001)
	42.3%	22.0%	18.2%	13.1%
		52.0%	43.0%	31.0%
RT campimetric test	.30 (.003)	−.13 (.229)	−.13 (.232)	−.05 (.621)
	9.0%	7.3%	7.3%	8.8%
		81.1%	81.1%	98.8%
Perimetric thresholds	− .71 (< .001)	−.60 (< .001)	−.64 (< .001)	−.66 ( < .001)
	50.4%	14.4%	9.5%	6.9%
		28.6%	18.8%	13.7%
R_Contrast thresholds	.47 ( < .001)	.35 ( < .001)	.40 ( < .001)	.44 ( < .001)
	22.1%	9.8%	6.1%	2.7%
		44.3%	27.6%	12.2%

For DPR thresholds, the correlation with age was slightly lower in the inner visual field (up to 10°) than in the outer visual field (10° – 20°). This reflected the steeper increase with age for the outer visual field. For DPR thresholds, we thus further extracted the influence of cognitive variables from correlations for the inner and outer ranges separately (Table 7) and found a strong reduction of shared variance for overt (saccadic exploration) and covert attention variables, but only minor effects for alertness and divided attention were extracted. For those cognitive variables that had an influence on the common variance between age and DPR thresholds, the effect was somewhat stronger for the outer parts of the DPR threshold map (Table 7).

**Table 7:** Correlations of DPR in the inner and outer visual field with age, and partial correlations where cognitive variables are extracted

Variable		Mean DPR Thresholds Inner Visual Field (0° – 10°)	Mean DPR Thresholds Outer Visual Field (10° – 20°)
Correlation with age *r* (*p*)		.52 ( < .001)	.59 ( < .001)
		27.0%	34.8%

Partial correlation variables extracted	Saccadic exploration mean RT	.23 (.031)	.34 (.001)
	Absolute extracted variance	21.8%	23.3%
	Relative extracted variance	80.7%	67.0%
	TAP Alertness median RT all trials	.47 ( < .001)	.54 ( < .001)
	Absolute extracted variance	5.0%	5.7%
	Relative extracted variance	18.5%	16.4%
	TAP Alertness mean RT difference cued-uncued trials	.53 ( < .001)	.60 ( < .001)
	Absolute extracted variance	−1.1%	−1.2%
	Relative extracted variance	−4.1%	−3.4%
	Divided attention median RT all trials	.48 ( < .001)	.56 ( < .001)
	Absolute extracted variance	4.0%	3.5%
	Relative extracted variance	14.8%	10.1%
	TAP Divided Attention mean number of correct detections	.49 ( < .001)	.57 ( < .001)
	Absolute extracted variance	3.0%	2.3%
	Relative extracted variance	11.1%	6.6%
	TAP Covert Attention (Posner paradigm) mean RT valid trials	.36 ( < .001)	.43 ( < .001)
	Absolute extracted variance	14.1%	16.3%
	Relative extracted variance	52.2%	46.8%
	TAP Covert Attention (Posner paradigm) mean RT invalid trials	.41 ( < .001)	.46 ( < .001)
	Absolute extracted variance	10.2%	13.7%
	Relative extracted variance	37.8%	39.4%
	TAP Covert Attention (Posner paradigm) mean RT difference valid−invalid trials	.45 ( < .001)	.50 ( < .001)
	Absolute extracted variance	6.8%	9.8%
	Relative extracted variance	25.2%	28.2%

Overall, age dependency of the topographical variables is partly mediated by attentional factors. The nontopographical variables of alertness and divided attention partly mediate age dependency of DPR thresholds and campimetric RTs, and less so perimetry, but not R_Contrast; overt attention mediates age dependency for all topographical variables except RTs; covert attention more strongly mediates the age dependency of all four topographical variables.

## Discussion

The first part of this study on visual field mapping over the life span had shown large differences between the visual field topographies of DPR thresholds, RT in campimetric testing, perimetric thresholds, and contrast thresholds of character recognition. In addition, the patterns of aging of those four main variables were markedly different, and we concluded that experimental or clinical testing that is limited to the fovea conceals important aspects of the development of visual functions over the life span. An earlier analysis of DPR thresholds in observers of the same sample (Poggel, Treutwein, et al., 2006) had further revealed a strong effect of the size of the attention focus on temporal resolution and an increasingly stronger effect of visual attention in the elderly. We therefore hypothesized that the topographies of the visual variables assessed in Part I of this study and their respective change over the life span not only reflect the functionality of the visual system per se, but also are influenced by higher visual and cognitive functions. In this second part of the Tölz Temporal Topography Study, we thus presented correlation patterns of the topographical variables introduced in Part I with a selected set of cognitive variables.

In the following, we will first relate our findings to the hypotheses stated in the introduction and to the literature before integrating the results from Part I and II of the study.

1. Influence of cognitive variables on visual field maps

Almost all variables of cognitive functions—alertness, divided attention, covert attention shift (Posner paradigm; Posner, 1980; Zimmermann & Fimm, 1995), and overt attention shift (saccadic RTs)—were correlated with the topographical variables measured in Part I. The correlations where higher for variables of temporal processing (DPR thresholds, RTs in campimetric testing) than for perimetric luminance and contrast thresholds of character recognition. In addition, a strong influence of the size of the attention focus had been shown for DPR thresholds (Poggel, Treutwein, et al., 2006). These results confirm the first part of our hypothesis—that is, that there is an influence of cognitive variables on visual field maps. Thus, the visual field map of a specific function is shaped not only by the properties of the visual system, but also by cognitive factors.

While there is extensive literature on the influence of cognitive variables on a wide range of perceptual performance measures, this study is, to our knowledge, the first to systematically show the influence of cognitive measures on the *topography* of visual field maps—in particular, of temporal functions. That spatial attention has an effect on (local) temporal resolution has been shown, for example, by Yeshurun and Levy (2003), who found a decrease of temporal resolution performance under attended conditions. Their paradigm, however, uses short-term cueing (i.e., transient attention; Nakayama & Mackeben, 1989), while our results stem from long-term monitoring of the visual field (i.e., from sustained attention). Our findings are also in accordance with a study by Wall, Woodward, and Brito (2004), who demonstrated reduced sensitivity in perimetric measurements with an increasing workload (and less attentional resources available for light detection) in healthy individuals.

The unusually large difference between invalid and valid trials of the covert attention test (Posner paradigm) that we found in our study may stem, in part, from the age of our observers, many of whom were much older than those in most cognitive psychological experiments. In addition, for some observers, there might have been a contamination with eye movements, which may have led to good RTs on valid trials, but longer RTs and an overall increased variance of RTs on invalid trials.

2. Differential influence of cognitive factors on visual field topography due to cognitive test demands

The measures of attentional functions selected here assess many aspects of the complex conglomerate of attention. Interestingly, the divided attention task in the TAP had only a weak connection with performance in the topographical tests. This is likely due to that the test assesses the ability to divide attention between modalities (visual and auditory), rather than *within* the visual modality (between different visual field locations or different visual subtasks). A purely visual test of divided attention might have been more strongly related to the topographical variables.

The highest among the correlations of cognitive with topographical variables (particularly those reflecting temporal processing) were those with the ability to overtly and covertly shift attention across the visual field. The variance of the topographical variables explained by RTs in the Posner paradigm or saccadic responses (Table 1) came close to the percentage explained by participant age (see Part I).

Attentional influences on temporal processing (Yeshurun, 2004; Yeshurun & Levy, 2003) and perimetric thresholds (Wall et al., 2004) have been reported by other authors, but to our knowledge, there has not been a systematic study relating different aspects of attention to visual field maps of different visual functions.

3. Differential influence of cognitive factors over the life span

All of the cognitive variables measured in the Tölz Temporal Topography Study systematically depended on the observer’s age. For most measures, young adults around 20 to 30 years of age showed the best performance, and over the life span performance significantly declined, with the oldest participants usually showing the lowest performance. Similarly, when the influence of overt attention (saccadic exploration) or covert attention (TAP Posner paradigm–spatial attention test), respectively, was extracted from the correlations of the topographical variables with age, the correlations were strongly reduced. Alertness and divided attention, in contrast, had only a small effect. Thus, a large part of the variation observed for the topographical variables over the life span seems not to stem from low-level visual factors, such as a deterioration of the visual system or of optical structures in the elderly, but rather, from top-down influences on visual processing.

4. Shape of visual field maps and their change over the life span depend on an interaction of visual functions and cognitive processes

For DPR thresholds, we further compared the correlations with age separately for the inner (< 10°) and outer (10° – 20°) visual fields. There was a slightly higher correlation for the outer than for the inner positions. Both correlations with age were reduced by partialling out cognitive factors, especially covert attention variables, again with a slightly stronger effect for the outer visual field positions. This effect and the influence of the size of the attention focus on temporal resolution mentioned above help to explain why the periphery “ages” somewhat more than the fovea and inner visual field with respect to DPR. Depending on the demands of a visual field measure on cognitive functions, cognitive aging may have differential effects on the visual field center and periphery.

The change of visual field topography over the life span is at least partly mediated by attentional factors. Alertness and divided attention partly mediate age dependency of DPR thresholds and campimetric RTs, but less so of perimetry, and not at all of R_Contrast. Partial correlation analysis revealed that the TAP subtests of *alertness* and *divided attention* were confounded with general RT measures. Hence, by the way these variables are measured in the TAP, they appear not to reflect purely cognitive function but are more influenced by visual RTs. This would explain their predominant mediation of the age dependency of RTs in the campimetric test. Due to the confoundedness with RT, the influence of the TAP measures *alertness* and *divided attention* is visible only in the topography of time-related variables like DPR thresholds and campimetric RTs.

In contrast, overt attention mediates age dependency for all topographical variables except RTs, and covert attention more strongly mediates the age dependency of all four topographical variables. Apparently, saccadic exploration (overt attention) and the Posner paradigm of the TAP (covert attention) reflect the ability to efficiently distribute and spatially shift around attentional resources in the visual field. Over the life span, these variables exert a strong influence on visual map topography because, along with the visual system, the cognitive resources and their flexibility are affected by aging.

In summary, cognitive variables—in particular, the size of the spatial focus of attention, as well as the ability of shifting it across the visual field, overtly and covertly—have a profound influence on visual field maps, particularly on the topography of temporal processing, and their change over the life span.

In Part I of this study we saw that observer age is not the most important predictor of visual performance in the topographical variables assessed here. Instead, the cognitive status of a person and the level of attentional capability may be more important for determining visual performance and the topography of visual field maps. First, there appears to be an influence of cognitive factors on the general level of performance—that is, the average across the visual field. Second, there are some aspects of attention selectively shaping the periphery and the center of the visual field. Attentional functions may be used to compensate for a deterioration of perceptual difficulties (Li & Lindenberger, 2002) due to reduced functionality of the optical and neural parts of the visual system with increasing age (Bellis, 1933; Birren & Fisher, 1995; Falkenstein et al., 2006; Haier et al., 2005; Owsley, Sekuler, & Siemsen, 1983; Plainis, Murray, & Chauhan, 2001; Schmidt, Galuske, & Singer, 1999; Spry & Johnson, 2001; Tyler & Hamer, 1993; Weale, 1963; see Schiefer et al., 2001, for a review).

## Conclusions

The Tölz Temporal Topography Study relates the topography of temporal and basic visual functions and their variation over the life span to higher-order visual parameters and to cognitive variables. Our data provide a reference system for developmental and neuropsychological studies (see Poggel, Treutwein, & Strasburger, 2011) and, for the first time, show systematic interactions between topographical patterns of visual (especially temporal) performance and cognitive factors across the life span. The study points to important aspects of assessing visual functions—for example, the importance of including the periphery of the visual field into functional characterization and the necessity of co-investigating the cognitive status of the observer, particularly in an elderly population. It seems that temporal processing encompasses various mechanisms (see the differences of visual field maps between RTs and DPR thresholds) and that these mechanisms are differentially affected by aging—that is, a reduced visual, cognitive, and general neural functionality.

## References

[CR1] Aizpurua A, Koutstaal W (2010). Aging and flexible remembering: Contributions of conceptual span, fluid intelligence, and frontal functioning. Psychology and Aging.

[CR2] Bellis CJ (1933). Reaction time and chronological age. Proceedings of the Society for Experimental Biology and Medicine.

[CR3] Birren JE, Fisher LM (1995). Aging and speed of behavior: Possible consequences for psychological functioning. Annual Review of Psychology.

[CR4] Brabyn J, Schneck M, Haegerstrom-Portnoy G, Lott L (2001). The Smith–Kettlewell Institute (SKI) longitudinal study of vision function and its impact among the elderly: An overview. Optometry and Vision Science.

[CR5] Bressler SL, Tang W, Sylvester CM, Shulman GL, Corbetta M (2008). Top-down control of human visual cortex by frontal and parietal cortex in anticipatory visual spatial attention. Journal of Neuroscience.

[CR6] Carrasco M (2006). Covert attention increases contrast sensitivity: Psychophysical, neurophysiological and neuroimaging studies. Progress in Brain Research.

[CR7] Castiello U, Umiltà C (1990). Size of the attentional focus and efficiency of processing. Acta Psychologica.

[CR8] Craik FI, Bialystok E (2006). Cognition through the lifespan: Mechanisms of change. Trends in Cognitive Sciences.

[CR9] Eckert MA, Keren NI, Roberts DR, Calhoun VD, Harris KC (2010). Age-related changes in processing speed: Unique contributions of cerebellar and prefrontal cortex. Frontiers in Human Neuroscience.

[CR10] Eimer M, Kiss M, Press C, Sauter D (2009). The roles of feature-specific task set and bottom-up salience in attentional capture: An ERP study. Journal of Experimental Psychology. Human Perception and Performance.

[CR11] Falkenstein M, Yordanova J, Kolev V (2006). Effects of aging on slowing of motor-response generation. International Journal of Psychophysiology.

[CR12] Fiorentini A, Porciatti V, Morrone MC, Burr DC (1996). Visual ageing: Unspecific decline of the responses to luminance and colour. Vision Research.

[CR13] Giesbrecht B, Woldorff MG, Song AW, Mangun GR (2003). Neural mechanisms of top-down control during spatial and feature attention. NeuroImage.

[CR14] Groth KE, Allen PA (2000). Visual attention and aging. Frontiers in Bioscience.

[CR15] Haegerstrom-Portnoy G, Schneck ME, Brabyn JA (1999). Seeing into old age: Vision function beyond acuity. Optometry and Vision Science.

[CR16] Haier RJ, Jung RE, Yeo RA, Head K, Alkire MT (2005). Structural brain variation, age, and response time. Cognitive, Affective, & Behavioral Neuroscience.

[CR17] Harvey LO (1997). Efficient estimation of sensory thresholds with ML-PEST. Spatial Vision.

[CR18] Kastner S, Pinsk MA, De Weerd P, Desimone R, Ungerleider LG (1999). Increased activity in human visual cortex during directed attention in the absence of visual stimulation. Neuron.

[CR19] Li KZ, Lindenberger U (2002). Relations between aging sensory/sensorimotor and cognitive functions. Neuroscience and Biobehavioral Reviews.

[CR20] Madden DJ (2007). Aging and visual attention. Current Directions in Psychological Science.

[CR21] Mangun GR, Buonocore MH, Girelli M, Jha AP (1998). ERP and fMRI measures of visual spatial selective attention. Human Brain Mapping.

[CR22] Mangun GR, Fannon SP (2007). Attention: Control in the visual cortex. Current Biology.

[CR23] Nakayama K, Mackeben M (1989). Sustained and transient components of focal visual attention. Vision Research.

[CR24] Natale E, Marzi CA, Girelli M, Pavone EF, Pollmann S (2006). ERP and fMRI correlates of endogenous and exogenous focusing of visual-spatial attention. European Journal of Neuroscience.

[CR25] Noesselt T., Hillyard S. A., Woldorff M. G., Schoenfeld A., Hagner T., Jancke L., Heinze H.-J. (2002). Delayed striate cortical activation during spatial attention. Neuron.

[CR26] Owsley C (2011). Aging and vision. Vision Research.

[CR27] Owsley C, Sekuler R, Siemsen D (1983). Contrast sensitivity throughout adulthood. Vision Research.

[CR28] Pesce C, Guidetti L, Baldari C, Tessitore A, Capranica L (2005). Effects of aging on visual attentional focusing. Gerontology.

[CR29] Plainis S, Murray IJ, Chauhan K (2001). Raised visual detection thresholds depend on the level of complexity of cognitive foveal loading. Perception.

[CR30] Poggel DA, Kasten E, Muller-Oehring EM, Bunzenthal U, Sabel BA (2006). Improving residual vision by attentional cueing in patients with brain lesions. Brain Research.

[CR31] Poggel DA, Treutwein B, Calmanti C, Strasburger H (2006). Increasing the temporal g(r)ain: Double-pulse resolution is affected by the size of the attention focus. Vision Research.

[CR32] Poggel DA, Treutwein B, Strasburger H (2011). Time will tell: Deficits of temporal-information processing in patients with visual field loss. Brain Research.

[CR33] Posner MI (1980). Orienting of attention. Quarterly Journal of Experimental Psychology.

[CR34] Rubin G. S., Bandeen-Roche K., Huang G. H., Munoz B., Schein O. D., Fried L. P. (2001). The association of multiple visual impairments with self-reported visual disability: SEE project. Investigative Ophthalmology & Visual Science.

[CR35] Schiefer U, Strasburger H, Becker ST, Vonthein R, Schiller J, Dietrich TJ, Hart W (2001). Reaction time in automated kinetic perimetry: Effects of stimulus luminance, eccentricity, and movement direction. Vision Research.

[CR36] Schmidt KE, Galuske RA, Singer W (1999). Matching the modules: Cortical maps and long-range intrinsic connections in visual cortex during development. Journal of Neurobiology.

[CR37] Schneck ME, Haegerstrom-Portnoy G, Lott LA, Brabyn JA, Gildengorin G (2004). Low contrast vision function predicts subsequent acuity loss in an aged population: The SKI study. Vision Research.

[CR38] Schneider KA, Kastner S (2009). Effects of sustained spatial attention in the human lateral geniculate nucleus and superior colliculus. Journal of Neuroscience.

[CR39] Soto D, Hodsoll J, Rotshtein P, Humphreys GW (2008). Automatic guidance of attention from working memory. Trends in Cognitive Sciences.

[CR40] Spear PD (1993). Neural bases of visual deficits during aging. Vision Research.

[CR41] Spry PG, Johnson CA (2001). Senescent changes of the normal visual field: An age-old problem. Optometry and Vision Science.

[CR42] Strasburger H. (1997). R_Contrast: Rapid measurement of recognition contrast thresholds. Spatial Vision.

[CR43] Toth LJ, Assad JA (2002). Dynamic coding of behaviourally relevant stimuli in parietal cortex. Nature.

[CR44] Travers S, West R (2008). Neural correlates of cue retrieval, task set reconfiguration, and rule mapping in the explicit cue task switching paradigm. Psychophysiology.

[CR45] Treutwein B (1989). Zeitliche aspekte der visuellen informationsverarbeitung.

[CR46] Treutwein B (1995). Minireview: Adaptive psychophysical procedures. Vision Research.

[CR47] Treutwein B (1997). YAAP: Yet another adaptive procedure. Spatial Vision.

[CR48] Treutwein B, Rentschler I (1992). Double pulse resolution in the visual field: The influence of temporal stimulus characteristics. Clinical Vision Science.

[CR49] Tyler CW, Hamer RD (1993). Eccentricity and the Ferry–Porter law. Journal of the Optical Society of America.

[CR50] Wall M, Woodward KR, Brito CF (2004). The effect of attention on conventional automated perimetry and luminance size threshold perimetry. Investigative Ophthalmology and Visual Science.

[CR51] Weale RA (1963). The aging eye.

[CR52] Werner JS, Peterzell DH, Scheetz AJ (1990). Light, vision, and aging. Optometry and Vision Science.

[CR53] West CG, Gildengorin G, Haegerstrom-Portnoy G, Schneck ME, Lott L, Brabyn JA (2002). Is vision function related to physical functional ability in older adults?. Journal of American Geriatrics Society.

[CR54] Yeshurun Y (2004). Isoluminant stimuli and red background attenuate the effects of transient spatial attention on temporal resolution. Vision Research.

[CR55] Yeshurun Y, Levy L (2003). Transient spatial attention degrades temporal resolution. Psychological Science.

[CR56] Zimmermann P, Fimm B (1995). Test Battery of Attention Performance (TAP).

